# Draft Genome Sequence of Alkalicoccus saliphilus DSM 15402^T^, a Haloalkaliphilic Bacterium Isolated from a Mineral Pool

**DOI:** 10.1128/MRA.00266-19

**Published:** 2019-06-13

**Authors:** Dacheng Qiu, Ziya Liao, Weidong Lu, Haisheng Wang, Jun Li, Baisuo Zhao

**Affiliations:** a Graduate School, Chinese Academy of Agricultural Sciences, Beijing, People’s Republic of China; b School of Life Sciences, Qingdao Agricultural University, Qingdao, People’s Republic of China; c Institute of Agricultural Resources and Regional Planning, Chinese Academy of Agricultural Sciences, Beijing, People’s Republic of China; University of Delaware

## Abstract

The haloalkaliphilic bacterium Alkalicoccus saliphilus DSM 15402^T^ was isolated from a mineral pool. It grows aerobically at an optimum of 15% (wt/vol) salinity and pH 9.0. The draft genome consists of approximately 3.52 Mb and contains 3,434 predicted genes. Various genes are potentially involved in the adaptation mechanisms for both osmotic stress and pH homeostasis, providing insight into specific adaptations to this double-extreme environment.

## ANNOUNCEMENT

The mesophilic haloalkaliphilic bacterium Alkalicoccus saliphilus DSM 15402^T^ was aerobically isolated from a mineral pool located in Malvizza in the Campania region of Italy (1). This isolate was originally characterized and recommended as Bacillus saliphilus and reclassified as Alkalicoccus saliphilus by Zhao et al. (2). Its growth occurs at a wide range of 1 to 25% (wt/vol) salinity (optimal salinity, 16%) and at pH 6.5 to 10.0 (optimal pH, 9.0) (2). To comprehend the adaptive strategies of survival under saline-alkaline conditions, the draft genome of *A. saliphilus* DSM 15402^T^ was sequenced using the Illumina MiSeq platform.

Cells of *A. saliphilus* grown under optimal conditions were collected (2), and genomic DNA was extracted by using the iTop microbial DNA isolation kit (Beijing, People’s Republic of China) according to the manufacturer’s instructions. An Illumina sequencing library was constructed using a TruSeq Nano DNA library kit with the whole-genome shotgun (WGS) method. Sequencing was performed at roughly 159× coverage with a paired-end read length of 2 × 300 bp. The filtered reads were quality inspected using Quake and the Burrows-Wheeler Aligner (BWA) with the default program parameters and *de novo* assembled into contigs using A5-miseq version 20150522 (3, 4). A total of 2,438,804 reads were yielded and assembled into 22 contigs. The total length of the draft genome sequence was 3,525,217 bp with a GC content of 45.69% and an *N*_50_ value of 561,230 bp. Automatic annotation was performed using the NCBI Prokaryotic Genome Annotation Pipeline (PGAP) (https://www.ncbi.nlm.nih.gov/genome/annotation_prok) and checked by the GenBank curation team for the standard requirements. Then, the genome files in GenBank format (gb file) were uploaded to the Integrated Microbial Genomes Expert Review (IMG ER) tool (https://img.jgi.doe.gov/cgi-bin/submit/main.cgi) for functional annotation after an analysis project identification (ID) number was registered in the GOLD Database (http://gold.jgi.doe.gov/index). Among the 3,434 predicted genes, 3,357 are potential protein-coding genes (CDSs). A total of 77 RNA genes (6 5S rRNAs, 4 16S rRNAs, 4 23S rRNAs, 59 tRNAs, and 4 other RNA genes) were predicted.

The genome sequence analysis revealed some crucial genes encoding putative proteins potentially associated with the adaptation mechanism of *A. saliphilus* to life under elevated salinity and alkaline pH. The genome harbors 1 gene cluster of *ectA*, *ectB*, and *ectC* genes for ectoine biosynthesis, 1 *glnA* gene for l-glutamine synthesis, 1 *betA* and 2 *betB* genes responsible for glycine betaine synthesis from choline, 3 genes for the glycine betaine-carnitine-choline transporter (BCCT family), and 9 genes for the glycine betaine ABC transporter. These genes show a strategy for coping with high external salinity by accumulating a large amount of compatible solutes, such as ectoine, glutamine, and glycine betaine (5, 6). Furthermore, the presence of 4 genes for the K^+^ uptake protein (Trk family) indicated that *A. saliphilus* also maintains osmotic balance by inducing a massive uptake of K^+^ when coping with a rapid osmotic shock (7). *A. saliphilus* is an obligate haloalkaliphile and therefore must have an adaptive strategy for pH homeostasis, because it has 4 genes of a monovalent cation:proton antiporter (CPA) belonging to the CPA 1 family (1 gene of the K^+^/H^+^ antiporter and 3 genes of the Na^+^/H^+^ antiporter), 7 genes of the multisubunit Na^+^/H^+^ antiporter, 1 gene of the Na^+^/H^+^ antiporter (NhaC family), and 8 genes for F_o_F_1_ ATP synthase (8–10). The genes on the genome of *A. saliphilus* that are identified in this report might play essential roles in the adaptive mechanisms of this haloalkaliphile.

### Data availability.

The draft genome sequence of Alkalicoccus saliphilus DSM 15402^T^ has been deposited at GenBank under the accession number PZJJ00000000. The draft genome sequence described in this paper is the first version (PZJJ01000000). The raw sequencing reads have been submitted to the Sequence Read Archive (SRA accession number SRR8449870) and are available in NCBI under BioProject number PRJNA437190 and BioSample number SAMN08640882.
